# Transcriptional feedback targeting Wnt pathway components reveals hidden heterogeneity in *Caenorhabditis elegans* seam cell lineages

**DOI:** 10.1093/genetics/iyag147

**Published:** 2026-06-12

**Authors:** Mar Ferrando-Marco, Simon Berger, Michalis Barkoulas

**Affiliations:** Department of Life Sciences, Imperial College, London SW7 2AZ, United Kingdom; Department of Molecular Life Sciences, University of Zurich, Zurich 8057, Switzerland; Department of Life Sciences, Imperial College, London SW7 2AZ, United Kingdom

**Keywords:** *C. elegans*, Wnt signaling, asymmetric cell division, symmetric cell division, seam cell, POP-1/TCF, PRY-1/Axin, SYS-1/β-catenin, feedback

## Abstract

Asymmetric cell division in the epidermal stem cells of *Caenorhabditis elegans*, known as seam cells, relies on the Wnt/β-catenin asymmetry pathway to generate daughter cells with distinct fates. However, whether components of this pathway are transcriptionally regulated during these divisions remains unclear. Here, we employ single-molecule fluorescence in situ hybridization to quantify mRNA distributions of key Wnt pathway components during L2 symmetric and asymmetric seam cell divisions. We find that transcripts encoding the negative regulators *pry-1*/Axin and *apr-1*/APC are enriched in posterior daughter cells, while those encoding the positive regulators *sys-1*/β-catenin, *wrm-1*/β-catenin, and *lit-1*/NLK, along with the transcription factor *pop-1*/TCF, are enriched in anterior daughter cells. Strikingly, molecular asymmetries are already evident following the L2 symmetric division, with anterior and posterior daughters exhibiting distinct levels of Wnt component expression and Wnt pathway activation. These mRNA distributions are surprising considering the established protein localizations that underpin the Wnt asymmetry model and suggest extensive postdivisional transcriptional regulation. We further demonstrate that *pop-1* and *pry-1* asymmetric expression partly depends on Wnt signaling activity. Investigation of protein distributions using knock-in reporters for PRY-1 and CAM-1 showed that protein accumulation patterns at L2 are consistent with transcript levels. Our findings uncover transcriptional feedback within the Wnt pathway that may reinforce robust fate specification and reveal molecular heterogeneity in seam cells with potential functional consequences for lineage behavior.

## Introduction

**Fig. 1.**
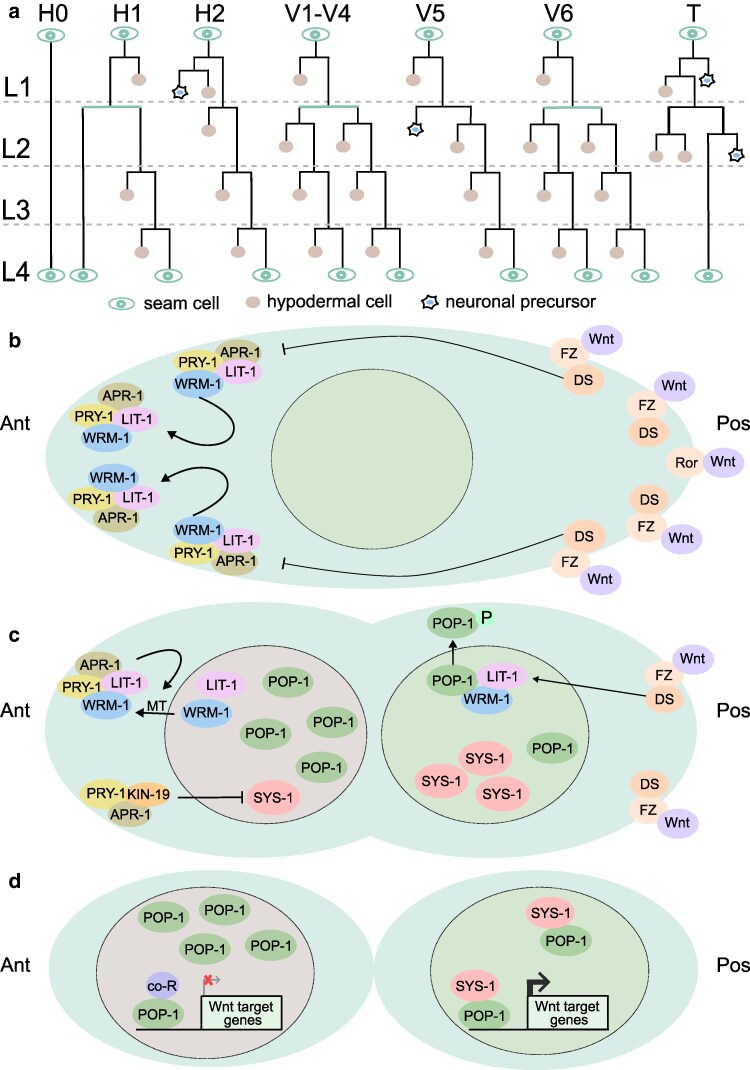
The Wnt/β-catenin asymmetry pathway controls the polarity of asymmetric seam cell divisions. a) Schematic showing seam cell divisions from L1 to L4. Seam cells are labeled in green, hypodermal cells are labeled in brown, and neuroblasts are labeled in blue. b) In the mother cell, positive regulators such as Frizzled (FZ) and Ror receptors as well as Dishevelled (DS) localize to the posterior cortex upon Wnt signal reception, while negative regulators APR-1/APC and PRY-1/Axin localize to the anterior cortex, recruiting WRM-1/β-catenin and LIT-1/NLK. Cortical WRM-1 further recruits APR-1 and WRM-1. c) In the dividing cell, cortical APR-1 stabilizes microtubules (MTs), facilitating the export of WRM-1 and LIT-1 from the anterior nucleus. APR-1, PRY-1, and KIN-19/CKI promote SYS-1/β-catenin degradation in anterior daughter cells, resulting in asymmetric localization toward the posterior nucleus. WRM-1 and LIT-1 phosphorylate POP-1/TCF in the posterior nucleus, triggering its nuclear export, with LIT-1 activity and localization dependent on MOM-4/MAPK and WRM-1. d) In anterior daughter cells, high nuclear POP-1 recruits corepressors (co-R) to repress Wnt target genes. In posterior daughter cells, low nuclear POP-1 forms a complex with SYS-1 to activate Wnt target gene expression.

The Wnt signaling pathway is an evolutionarily conserved cascade that controls cell polarity and fate decisions in the context of asymmetric cell division across diverse organisms (Bielen and Houart 2014; Lam and Phillips 2017; Habib and Acebrón 2022). Asymmetric cell division is a fundamental mechanism by which stem and progenitor cells generate cellular diversity, producing 1 daughter that maintains stem/progenitor fate and 1 that differentiates. Maintaining an appropriate balance between self-renewal and differentiation is essential for tissue homeostasis (Morrison and Kimble 2006; Knoblich 2008). In *Caenorhabditis elegans*, the lateral epidermal seam cells provide a powerful model for investigating Wnt-dependent control of cell polarity and fate determination. These cells are arranged in 2 bilateral rows running along the anterior–posterior axis and undergo a stereotypical series of symmetric and asymmetric divisions during larval development, generating most of the syncytial epidermis and certain neuronal precursor cells (Fig. 1a) (Sulston and Horvitz 1977; Joshi et al. 2010; Chisholm and Hsiao 2012). During asymmetric divisions, the posterior daughter typically retains seam cell identity and continues dividing in subsequent larval stages, whereas the anterior daughter exits the stem cell-like program, differentiates and fuses with the surrounding hyp7 syncytium. A single symmetric division in selected seam cell lineages at the L2 stage expands the seam cell population from 10 to 16 per lateral side, a number that is highly consistent in wild-type populations (Boukhibar and Barkoulas 2016; Katsanos et al. 2017; Koneru et al. 2021). Following the final larval stage, seam cells undergo terminal differentiation, fuse into a syncytium, and secrete the cuticular ridges known as alae.

The Wnt/β-catenin asymmetry pathway plays a central role in this process by globally regulating the fate of seam cell daughters: reduced Wnt signaling causes both daughters to adopt differentiated fates, while constitutive pathway activation leads to symmetric retention of the seam cell fate and supernumerary seam cells (Gleason and Eisenmann 2010; Gorrepati et al. 2013). In this modified version of the Wnt pathway, Wnt component localization and protein stability are tightly regulated to generate asymmetric protein distribution between daughter cells (Takeshita and Sawa 2005; Mizumoto and Sawa 2007a, 2007b; Phillips et al. 2007), leading to differentiation of the anterior daughter cell and retention of seam cell fate in the posterior daughter cell. In the dividing mother cell, Wnt ligands promote the posterior cortical localization of Frizzled and Ror receptors together with Dishevelled proteins, while the negative regulators APR-1/Adenomatous Polyposis Coli (APC) and PRY-1/Axin localize to the anterior cortex (Fig. 1b) (Goldstein et al. 2006; Mizumoto and Sawa 2007b; Green et al. 2008; Baldwin and Phillips 2014). At the anterior cortex, APR-1 recruits WRM-1/β-catenin, which in turn recruits the kinase LIT-1/Nemo-Like Kinase (NLK), establishing a feed-forward loop that reinforces cortical asymmetry (Fig. 1b) (Takeshita and Sawa 2005; Mizumoto and Sawa 2007a, 2007b; Sugioka et al. 2011). During division, APR-1 also promotes the export of WRM-1 and LIT-1 from the anterior nucleus through regulation of microtubule dynamics and, together with KIN-19/Casein Kinase 1 alpha (CK1&alpha;), promotes degradation of SYS-1/β-catenin (Fig. 1c) (Mizumoto and Sawa 2007b; Banerjee et al. 2010; Sugioka et al. 2011; Baldwin and Phillips 2014). In posterior daughter cells, the absence of anterior negative regulators allows stabilization of SYS-1 and nuclear retention of WRM-1 and LIT-1. High nuclear WRM-1 promotes activation of LIT-1 leading to phosphorylation of the TCF transcription factor POP-1 and its nuclear export (Fig. 1c) (Meneghini et al. 1999; Lo et al. 2004; Takeshita and Sawa 2005; Yang et al. 2011, 2015). The resulting ratio of nuclear levels of POP-1 and SYS-1 is thought to determine transcriptional output in the daughter cells, with low POP-1 and high SYS-1 in posterior daughters promoting transcriptional activation, and high POP-1 and low SYS-1 in anterior daughters favoring transcriptional repression (Fig. 1d) (Huang et al. 2007; Phillips et al. 2007; Bekas and Philips 2022).

To date, the Wnt/β-catenin asymmetry pathway has been characterized primarily through analyses of protein localization through translational reporters imaged at early stages. Therefore, to what extent components of this pathway are also regulated transcriptionally during division remains unclear. Here, we use single-molecule fluorescence in situ hybridization (smFISH) to provide a comprehensive transcriptional analysis of key Wnt/β-catenin asymmetry pathway components in V1 to V4 seam cell lineages, which show identical division patterns. We focus on the L2 stage to capture both symmetric and asymmetric division events. We uncover unexpected asymmetric mRNA expression patterns following division, with the negative regulators *pry-1* and *apr-1* enriched in posterior seam-fated daughters and the positive regulators *sys-1*, *wrm-1*, and *lit-1*, together with the transcription factor *pop-1*, enriched in anterior differentiating daughters. Notably, we find that daughters generated from seemingly symmetric divisions differ significantly in the distributions of Wnt components and in their capacity for Wnt pathway activation, indicating that they are not truly identical and harbor substantial molecular heterogeneity. These transcriptional patterns were largely not anticipated based on the known protein distributions, and we demonstrate that they are likely to reflect transcriptional feedback in the pathway. We propose that transcriptional feedback may contribute to robust seam cell fate determination and influence how cells within a seam cell lineage respond to genetic or environmental changes.

## Materials and methods

### *C. elegans* maintenance and genetics

All *C. elegans* strains in this study were maintained at 20 °C on nematode growth medium (NGM) plates seeded with *Escherichia coli*  OP50 (Brenner 1974). A list of strains used in this study is provided in Supplementary Table 1.

### RNA interference feeding

RNAi by feeding was performed using *E. coli*  HT115 expressing double-stranded RNA targeting *egl-18*, as previously described (Kamath and Ahringer 2003; Kamath et al. 2003). Bacterial cultures were grown overnight, and 300 μL were seeded onto NGM plates containing 25 μg/mL ampicillin, 12.5 μg/mL tetracycline, and 1 mM IPTG. RNAi treatment was performed during postembryonic development by seeding eggs of the indicated strains directly onto *egl-18* RNAi plates. The *egl-18* RNAi clone used in this study was obtained from the Ahringer RNAi Library (Kamath and Ahringer 2003) (Source Bioscience).

### Single-molecule fluorescent in situ hybridization

Animals were synchronized by bleaching and were fixed at 25 h postbleaching 4% formaldehyde for 45 min and permeabilized with 70% ethanol. Samples were then incubated with a pool of 24 to 48 oligos labeled with Quasar 670 (Biosearch Technologies, Novato, CA, USA) for 16 h. Imaging was performed using the 100× objective of an epifluorescence Ti-eclipse microscope (Nikon) and DU-934 CCD-17291 camera (Andor Technology) operated via the Nikon NIS Elements Software. Twenty-three z-stack slices of 0.8 μm were acquired for each animal. Spot quantification was performed using MATLAB (MathWorks) as previously described (Raj et al. 2008). For smFISH quantification, regions of interest matching the eye-shaped cells were manually drawn around each seam cell guided by the *scm::GFP* (nuclear) and AJM-1::GFP (membrane) fluorescence and blinded to the smFISH signal. Only puncta within the region of interest (ROI) were included in the quantification. Z-slices containing spots were max-projected to give a single image, inverted, and merged to the GFP channel using ImageJ (NIH). A complete list of the smFISH probes used in this study is provided in Supplementary Table 1.

### Confocal microscopy

Protein reporter images were acquired using a Leica SP8 Inverted confocal microscope with a 63×/1.4 oil differential interference contrast (DIC) objective controlled by LAS X software. Animals were mounted in 5% agarose pads containing 100 μM sodium azide (NaN_3_) and secured with a coverslip. To visualize L2 symmetric and asymmetric divisions, animals were observed at 26 h postbleaching. Z-stack slices of 0.4 μm were acquired, and 7 slices were SUM projected for quantification using Fiji/ImageJ. For each seam cell daughter, membrane-associated PRY-1::mNeonGreen signal was quantified using a segmented line selection manually traced along the cell cortex. The membrane ROI was defined using the corresponding DIC image to accurately identify the cell boundary, and this selection was then applied to the mNeonGreen channel. The line width was set to fully encompass membrane-associated PRY-1 signal, including punctate cortical accumulations, while minimizing contribution from the cytoplasm. Conversely, cytoplasmic PRY-1::mNeonGreen signal was quantified using a manually defined area selection drawn within the cell boundaries based on DIC. Cytoplasmic ROIs were selected based on cell morphology without explicit nuclear exclusion and positioned away from the cell cortex to minimize contribution from membrane-associated signal. For both measurements, fluorescence intensity was quantified as corrected total cell fluorescence (CTCF), calculated as:


CTCF=integrateddensity−(ROIarea×meanbackgroundintensity).

Background fluorescence was measured for each image using 3 regions of interest placed in signal-free areas outside the animal, and the average background value was used for correction. All images were acquired and analyzed using identical settings.

The data was normalized (rescaled) to [0,1] using the formula:


$$z=\;\frac{x\;-\;\min(X)}{\max(X)\;-\;\min(X)}$$

where *X* is the data, *x* is a datapoint, and *z* is the corresponding rescaled datapoint. Picture editing was performed using straightening tool and stitching macro from FIJI (Preibisch et al. 2009).

### Microfluidic imaging

Microfluidic imaging was performed according to (Berger et al. 2021, 2025). Briefly, NA22 bacteria were grown overnight in 40 mL LB with shaking at 37 °C to an OD600 of ∼1.9, washed 3 times with S-basal, and finally resuspended in ∼1 mL S-basal. Finally, 1 mL of bacterial suspension was mixed with 0.65 mL OptiPrep density gradient medium and 0.33 mL S-basal supplemented with 1% Pluronic F127 and finally filtered using a 10-µm strainer.

Animals were synchronized by bleaching, and embryos were allowed to hatch overnight in M9, arresting at the L1 larval stage. For imaging at late L1, animals were plated on NGM plates, grown for 16 h, and collected using S-basal.

Animals were then loaded into an L1 to L4 microfluidic device and imaged at 15-min intervals for 24 h using an upright microscope (DMRA2, Leica), equipped with a sCMOS camera (Prime BSI, Photometrics), an LED combiner (Spectra, Lumencor), and a piezo objective drive (MIPOS 100, Piezosystems Jena). Z-stacks of 0.5 μm were acquired using a 40× oil objective. Image acquisition was controlled using custom-built MATLAB scripts (MATLAB 2023b, MathWorks) and custom-built microcontrollers (Arduino Mega 2560) for coordination of fluorescence and bright-field LEDs, piezo, and camera. All images were acquired at 20 ± 0.5 °C, with temperature control achieved either via a microscope cage incubator (H201-T-UNIT-BL-CRYO and H201-ENCLOSURE-CRYO, Okolab).

POP-1 and HMG-helper optimal promoter (POPHHOP) expression was scored manually as the presence or absence of detectable nuclear GFP signal above background in individual seam cells at each time point. Images were deconvolved using the YacuDecu implementation of CUDA-based Richardson Lucy deconvolution in MATLAB (www.github.com/bobpepin/YacuDecu) with point spread functions (PSFs) generated using microPSF (Li et al. 2017), and images were edited for presentation using Fiji/ImageJ.

### Statistical analysis

Graphic representation and statistical analysis were performed using R. Error bars in all graphs represent 95% confidence intervals. Unless otherwise indicated, statistical significance was assessed using a 2-tailed Welch's *t*-test. In most datasets, comparisons were defined a priori and limited to single pairwise tests. Therefore, multiple comparison correction was not applied. For experiments involving multiple comparisons on the same dataset (Fig. 6, c and d), the Bonferroni correction was used to control the risk of Type I errors. To account for the fact that multiple cells were measured per animal, additional analyses were performed focusing this time on the animal level and averaging measurements per animal. In this case, because of the more limited number of animals per condition, statistical significance was assessed using bootstrap resampling, which does not rely on distributional assumptions. Full details of all statistical results are provided in Supplementary Table 2. Results were considered statistically significant when *P* < 0.05. Asterisks indicate significance as follows: *P < 0.05, **P < 0.01, ***P < 0.005, and ****P < 0.001.

## Results

### Negative regulators *pry-1* and *apr-1* are enriched in posterior daughter cells

**Fig. 2.**
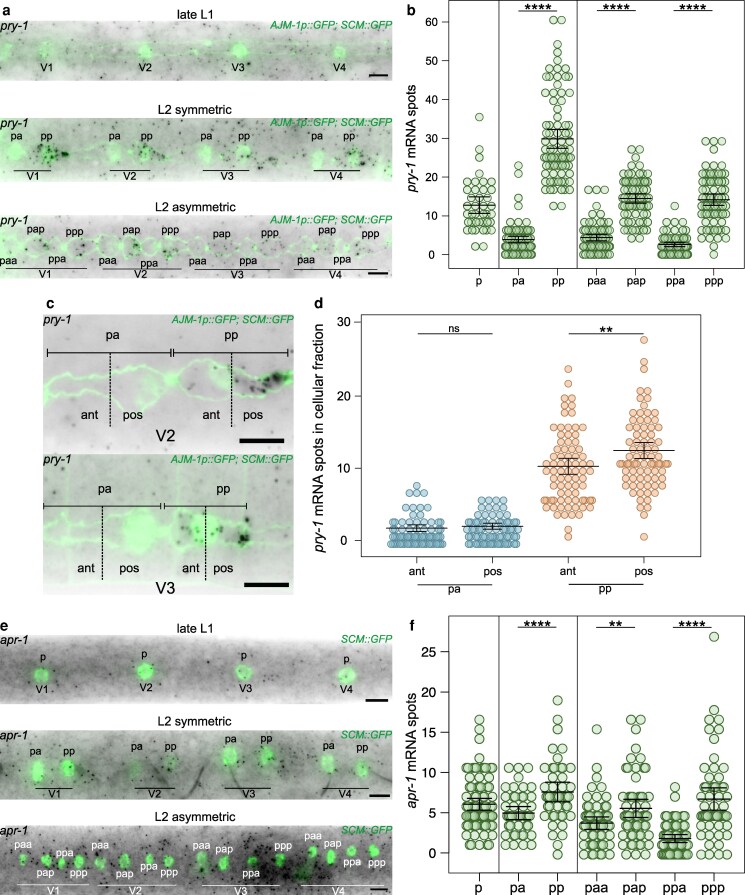
Negative regulators *pry-1* and *apr-1* are enriched in posterior daughter cells. a) Representative *pry-1* smFISH images at late L1, and following the L2 symmetric and L2 asymmetric divisions. b) Quantification of *pry-1* mRNA spots in V1-V4 seam cells before (p) and after the L2 symmetric division (pa, pp) and following the L2 asymmetric division (paa, pap, ppa, ppp); 40 ≤ *n* ≤ 84 cells per condition. c) Detailed image of *pry-1* smFISH following the L2 symmetric division showing enrichment of mRNAs in the posterior (pos) fraction of the cell compared with the anterior (ant) fraction. Anterior and posterior fractions correspond to half of the total length of the cell. d) Quantification of *pry-1* smFISH spots in the anterior and posterior fractions of pa and pp cells following the L2 symmetric division. *n* = 72 pa cells and 79 pp cells. e) Representative *apr-1* smFISH images at late L1 and following the L2 symmetric and L2 asymmetric divisions. f) Quantification of *apr-1* mRNA spots in V1 to V4 seam cells before (p) and after the L2 symmetric division (pa, pp) and following the L2 asymmetric division (paa, pap, ppa, ppp); 44 ≤ *n* ≤ 80 cells per condition. In a), c), and e), seam cell nuclei are labeled using *SCM::GFP*, and in a) and c), seam cell membrane with the apical junction marker *ajm-1p::ajm-1::GFP.* Scale bars are 5 μm in a), c), and e). Error bars in b), d), and f) show the mean ± 95% confidence intervals and ****P < 0.001 and **P < 0.01 with a 2-tailed Welch's *t*-test.

To characterize the expression pattern of Wnt/β-catenin asymmetry pathway components in seam cells, we performed smFISH focusing on V1 to V4 seam cells at late L1 and L2 stages. We first examined the expression patterns of negative regulators *pry-1* and *apr-1* (Fig. 2). Analysis of *pry-1* mRNA distribution at late L1 revealed expression in seam cells (p) prior to the L2 symmetric division (Fig. 2, a and b). Interestingly, following the L2 symmetric division, posterior (pp) cells displayed significantly higher *pry-1* mRNA levels compared with anterior (pa) cells (Fig. 2b). The same pattern was observed following the L2 asymmetric division, where posterior daughter cells (pap and ppp) exhibited significantly higher *pry-1* mRNA levels compared with their corresponding anterior sister cells (paa and ppa) (Fig. 2b). Quantification of the frequency of cells displaying intense spots in the nucleus that is indicative of active transcription supported a repression in anterior cells (pa, paa, and ppa) (Supplementary Fig. 1a). We also quantified *pry-1* mRNA distribution within individual cells and found that *pry-1* transcripts were enriched in the posterior fraction of pp cells relative to their anterior fraction (Fig. 2, c and d). This suggests that in the case of *pry-1*, asymmetric localization of mRNAs may contribute to asymmetric inheritance in daughter cells. Similarly, *apr-1* mRNA expression was enriched in posterior daughter cells following both L2 symmetric and asymmetric divisions (Fig. 2, e and f). Taken together, these results suggest enrichment of negative regulators of Wnt signaling in the posterior seam cells likely due to repression of expression in anterior cells.

### Positive regulators *sys-1*, *wrm-1*, and *lit-1* are enriched in anterior daughter cells

**Fig. 3.**
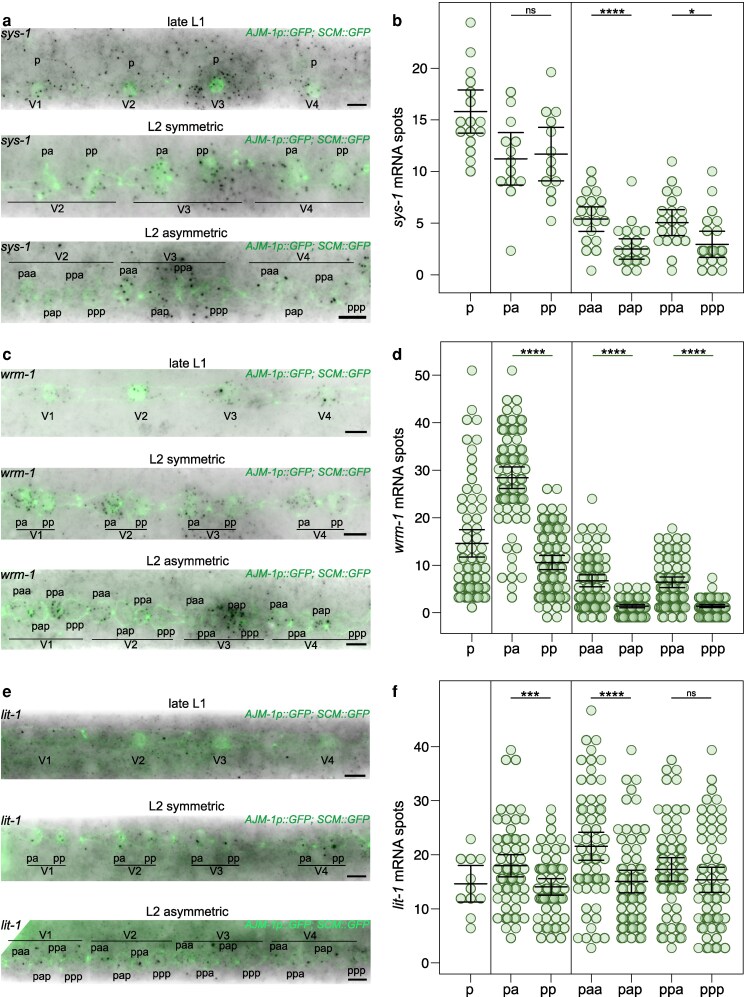
Positive regulators *sys-1*, *wrm-1*, and *lit-1* are enriched in anterior daughter cells. a) Representative *sys-1* smFISH images at late L1 and following the L2 symmetric and L2 asymmetric divisions. b) Quantification of *sys-1* mRNA spots in V1-V4 seam cells before (p) and after the L2 symmetric division (pa, pp) and following the L2 asymmetric division (paa, pap, ppa, ppp); 13 ≤ *n* ≤ 22 cells per condition. c) Representative *wrm-1* smFISH images at late L1 and following the L2 symmetric and L2 asymmetric divisions. d) Quantification of *wrm-1* mRNA spots in V1-V4 seam cells before (p) and after the L2 symmetric division (pa, pp) and following the L2 asymmetric division (paa, pap, ppa, ppp); 67 ≤ *n* ≤ 79 cells per condition. e) Representative *lit-1* smFISH images at late L1 and following the L2 symmetric and L2 asymmetric divisions. f) Quantification of *lit-1* mRNA spots in V1 to V4 seam cells before (p) and after the L2 symmetric division (pa, pp) and following the L2 asymmetric division (paa, pap, ppa, ppp); 11 ≤ *n* ≤ 63 cells per condition. In a), c), and e), seam cell nuclei are labeled using *SCM::GFP* and seam cell membrane with the apical junction marker *ajm-1p::ajm-1::GFP*, and scale bars are 5 μm. Error bars in b), d), and f) show the mean ± 95% confidence intervals and ****P < 0.001, ***P < 0.005, and *P < 0.05 with a 2-tailed Welch's *t*-test.

We next examined the expression pattern of positive regulators of the Wnt β-catenin asymmetry pathway, namely the β-catenins *sys-1* and *wrm-1*, and the kinase *lit-1*. *sys-1* smFISH analysis revealed expression in seam cells at late L1 (Fig. 3, a and b). Following the L2 symmetric division, *sys-1* mRNA levels remained comparable between pa and pp cells (Fig. 3, a and b), whereas following the L2 asymmetric division, anterior daughters (paa and ppa) showed significantly higher *sys-1* mRNA compared with their posterior sisters (pap and ppp) (Fig. 3b). *wrm-1* mRNA levels were significantly higher in anterior daughters compared with posterior daughters following both symmetric and asymmetric divisions (Fig. 3, c and d). Following the L2 asymmetric cell division, posterior daughter cells presented a strong reduction in *wrm-1* transcript abundance, with 32% of cells showing no *wrm-1* mRNA at all (Fig. 3d). Quantification of the frequency of cells displaying intense smFISH spots in the nucleus also supported a potential repression in posterior daughter cells, although active *wrm-1* transcription in anterior cells decreased over time (Supplementary Fig. 1b). *lit-1* showed similar expression across L1 and L2 stages and mild enrichment in anterior daughter cells following the L2 divisions (Fig. 3, e and f). Taken together, these results suggest that positive regulators, with *wrm-1* being the most striking example, show enrichment in anterior cells following division likely due to repression in posterior cells.

### *pop-1* expression is enriched in anterior daughter cells

**Fig. 4.**
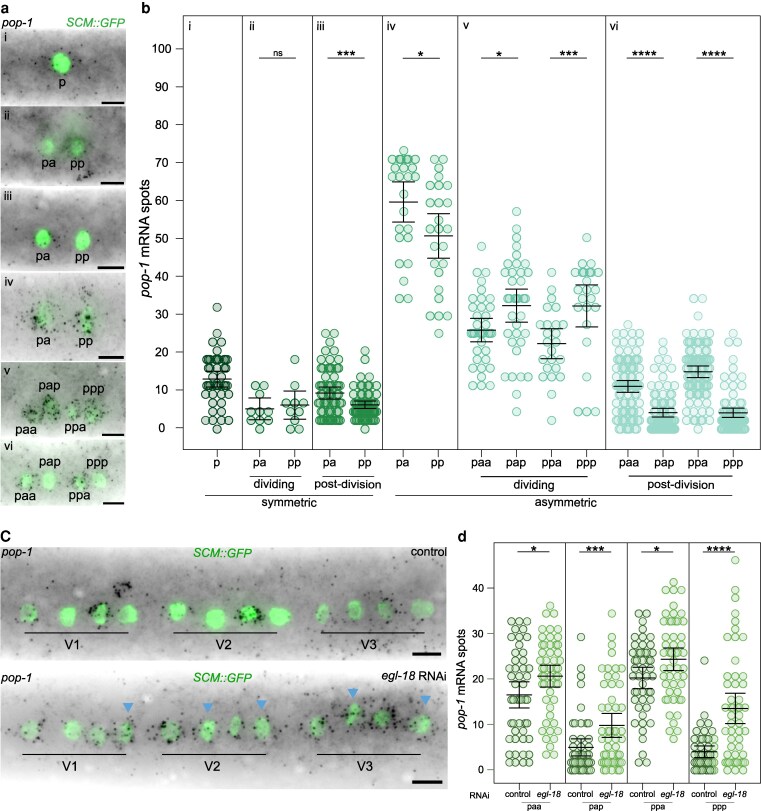
*pop-1* expression is enriched in anterior daughter cells. a) Representative *pop-1* smFISH images from the late L1 stage to after the L2 asymmetric division. i) late L1, ii) during L2 symmetric division, iii) after L2 symmetric division, iv) before L2 asymmetric division, v) anaphase–telophase of L2 asymmetric division, and vi) after the L2 asymmetric division. b) Quantification of *pop-1* mRNA spots in V1 to V4 lineages in each of the developmental stages shown in a) (9 ≤ *n* ≤ 87 cells per condition). Following symmetric and asymmetric divisions, anterior daughter cells (pa, paa, and ppa) showed higher number of *pop-1* mRNA spots compared with corresponding posterior daughter cells (pp, ppa, and ppp). c) Representative *pop-1* smFISH images following the L2 asymmetric division upon *egl-18* RNAi vs control treatment. Arrowheads point to posterior daughter cells in *egl-18* RNAi-treated animals showing increased levels of *pop-1* transcripts compared with control. d) Quantification of *pop-1* mRNA spots in V1-V4 lineages in anterior and posterior daughter cells following the L2 asymmetric division upon *egl-18* RNAi vs control treatment (48 ≤ *n* ≤ 51 cells per condition). Error bars in b) and d) show the mean ± 95% confidence intervals and ****P < 0.001, **P < 0.01, and * *P* < 0.05 with a 2-tailed Welch's *t*-test. In a) and c), seam cells are labeled using *SCM::GFP*, and scale bars are 5 μm.

These unexpected patterns in the localization of Wnt machinery suggested a high degree of transcriptional regulation and led us to investigate in more detail the mRNA pattern of the transcription factor POP-1. POP-1 shows a well-described anterior nuclear enrichment at the protein level in daughter cells following asymmetric division (Phillips and Kimble 2009), as well as a newly reported anterior enrichment at the transcript level (Ferrando-Marco and Barkoulas 2025). At late L1, seam cells showed *pop-1* mRNA expression that served as the baseline (Fig. 4, a-i and b). During the L2 symmetric division*, pop-1* expression was maintained at similar levels between daughter cells (Fig. 4, a-ii and b). Once the L2 symmetric division was completed, anterior daughter cells (pa) exhibited significantly higher *pop-1* mRNA compared with posterior daughters (pp) (Fig. 4, a-iii and b). Prior to the L2 asymmetric division, *pop-1* expression levels increased dramatically (Fig. 4, a-iv and b), and the difference between anterior–posterior cells (pa vs pp) was maintained. During anaphase–telophase of the L2 asymmetric division, *pop-1* mRNA appeared slightly enriched in posterior daughters (Fig. 4, a-v and b). However, following completion of the L2 asymmetric division, posterior daughter cells (pap and ppp) showed markedly lower *pop-1* mRNA levels compared with anterior daughters (paa and ppa), with 33% of cells showing no *pop-1* mRNAs (Fig. 4, a-vi and b), suggesting repression in posterior cells.

To assess whether *pop-1* asymmetric expression is sensitive to pathway output, we knocked down the Wnt downstream effector and POP-1 target *egl-18* using RNAi. This treatment is known to result in a decrease in the terminal seam cell number because of failure of posterior cells to maintain the seam cell fate (Gorrepati et al. 2013; Ferrando-Marco and Barkoulas 2025). *egl-18* RNAi-treated animals showed a significant increase in *pop-1* expression in both anterior and posterior daughter cells, with the increase in posterior cells being more striking (Fig. 4, c and d). These results suggest that the asymmetric *pop-1* expression pattern is at least partially dependent on EGL-18 function.

**Fig. 5.**
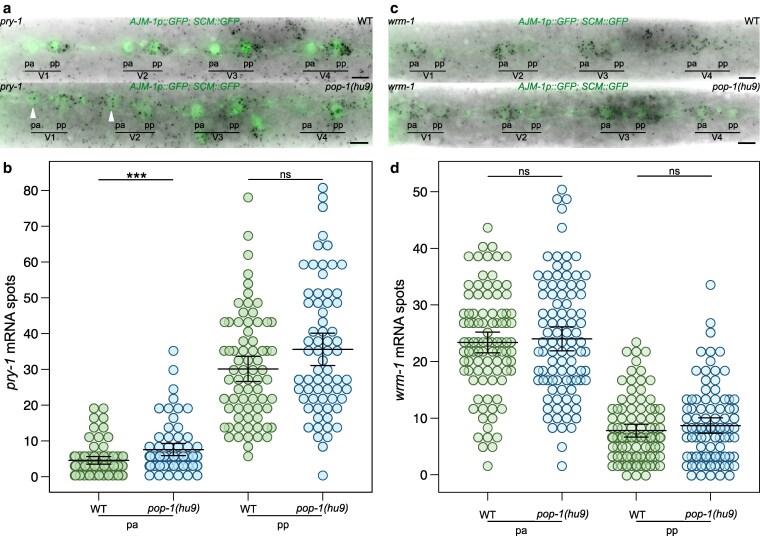
*pop-1(hu9)* affects the asymmetric expression of *pry-1* but not *wrm-1*. a) Representative *pry-1* smFISH images following the L2 symmetric division in wild-type and *pop-1(hu9)* animals. White arrows point to anterior daughter cells showing increased *pry-1* expression. b) Quantification of *pry-1* mRNA spots in anterior (pa) and posterior (pp) seam cells following the L2 symmetric division in wild-type and *pop-1(hu9)* animals; 67 ≤ *n* ≤ 72 cells per condition. c) Representative *wrm-1* smFISH images following the L2 symmetric division in wild-type and *pop-1(hu9)* animals. d) Quantification of *wrm-1* mRNA spots in anterior (pa) and posterior (pp) seam cells following the L2 symmetric division in wild-type and *pop-1(hu9)* animals; 94 ≤ *n* ≤ 95 cells per condition. In a) and c), seam cell nuclei are labeled using *SCM::GFP* and seam cell membrane with the apical junction marker *ajm-1p::ajm-1::GFP*. Scale bars are 5 μm. Error bars in b) and d) show the mean ± 95% confidence intervals and ***P < 0.005 with a 2-tailed Welch's *t*-test.

To test more directly whether asymmetric expression of Wnt pathway components depends on POP-1, we analyzed *pry-1* and *wrm-1* expression in the *pop-1(hu9)* hypomorph. In this mutant background, seam cell patterning is not disrupted during the L2 developmental stage, but anterior cells can retain the seam cell fate instead of differentiating following the L4 division (Ferrando-Marco and Barkoulas 2025). As expected, in wild-type animals, *pry-1* transcripts were significantly enriched in posterior daughters (pp) relative to anterior daughters (pa) following the L2 symmetric division. In *pop-1(hu9)* animals, posterior enrichment was maintained, but *pry-1* transcript levels were significantly increased in anterior daughters, resulting in a reduced difference between pa and pp cells (Fig. 5, a and b). No significant difference was observed between wild-type and *pop-1(hu9)* animals in the case of *wrm-1* expression (Fig. 5, c and d). These results suggest that POP-1 function can contribute to the regulation of asymmetric Wnt component expression.

### Daughter cells resulting from symmetric and asymmetric divisions show different levels of PRY, CAM-1, and Wnt signaling activity

**Fig. 6.**
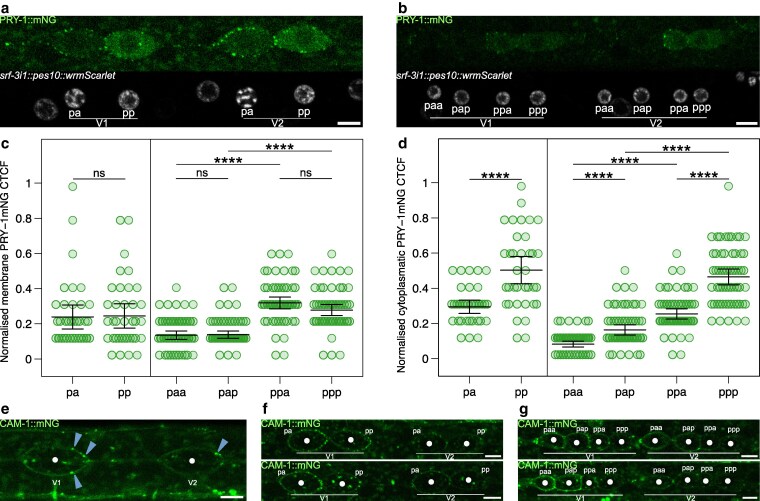
Analysis of PRY-1 and CAM-1 expression following symmetric and asymmetric divisions shows asymmetric protein localization between daughter cells. a and b) Representative PRY-1::mNeonGreen images before a) and after b) the L2 asymmetric division. Images are SUM projections of 7 z-stack slices acquired at 0.4 μm intervals. Seam cell nuclei is labeled with *srf-3i::pes-10::wrmScarlet*. c and d) Quantification of PRY-1::mNeonGreen fluorescence intensity following the L2 symmetric (pa, pp) and asymmetric (paa, pap, ppa, ppp) divisions in the membrane c) and in the cytoplasm d). Fluorescence intensities were measured as continuous CTCF values and normalized to a 0 to 1 scale. Error bars show the mean ± 95% confidence intervals and ****P < 0.001 with a 2-tailed Welch's *t*-test. For L2 asymmetric data, *P*-values were adjusted using Bonferroni correction, 36 ≤ *n* ≤ 60 cells per condition. e to g) Representative CAM-1::mNeonGreen images at late L1 e) and following the L2 symmetric f), and asymmetric g) divisions. Dots mark mark the approximate center of each cell to aid identification. Arrows indicate posterior membrane-localized foci. Scale bars are 5 μm in a), b), e), f), and g).

Next, we asked whether the unanticipated differential expression of Wnt signaling components following symmetric and asymmetric division may influence distributions at the protein level. We first chose to examine PRY-1 protein localization using a PRY-1::mNeonGreen knock-in strain (Heppert et al. 2018), as differences in *pry-1* mRNA expression in cell daughters following symmetric and asymmetric divisions were among the most striking observed (Fig. 2b). We found that prior to the L2 asymmetric division, PRY-1::mNeonGreen showed anterior membrane localization in both pa and pp cells as expected, but pp cells displayed higher cytoplasmic PRY-1 compared with pa cells (Fig. 6, a to d). Following the L2 asymmetric division, daughter cells from the posterior branch (ppa and ppp) displayed significantly higher PRY-1::mNeonGreen fluorescence intensity in both the membrane and cytoplasm compared with the daughters of the anterior branch (paa and pap) (Fig. 6, b to d). The higher PRY-1 levels observed in the posterior branch after the asymmetric division may arise from the earlier transcript asymmetry seen in the mother cell by smFISH. In addition, posterior daughter cells (pap and ppp) showed significantly higher levels of cytoplasmic PRY-1 compared with their respective anterior daughter cells (paa and ppa), which is consistent with the smFISH data.

We then focused on the expression of the Ror receptor CAM-1, for which we previously reported that *cam-1* mRNA is enriched in anterior daughter cells following both symmetric and asymmetric divisions (Ferrando-Marco et al. 2026). We used a CAM-1::mNeonGreen knock-in strain (Heppert et al. 2018) to study protein localization. At late L1, mother cells showed posterior membrane localization as previously reported for other Wnt receptors (Mizumoto and Sawa 2007a, 2007b). We focused on V1 cells because, across all animals analyzed, CAM-1 signal was consistently stronger in the V1 lineage compared with V2 to V4, in agreement with what we previously reported for *cam-1* mRNA (Ferrando-Marco et al. 2026). Following symmetric division, 57% animals analyzed showed a clear enrichment of CAM-1 at the posterior cortex of anterior daughter cells (pa) compared with posterior daughter cells in the V1 lineage (Fig. 6f). Following asymmetric division, 80% animals analyzed displayed higher membrane-localized CAM-1 in the anterior branch (paa and pap) relative to daughter cells from the posterior branch (ppa and ppp) (Fig. 6g), which is consistent with the smFISH data (Ferrando-Marco et al. 2026).

**Fig. 7.**
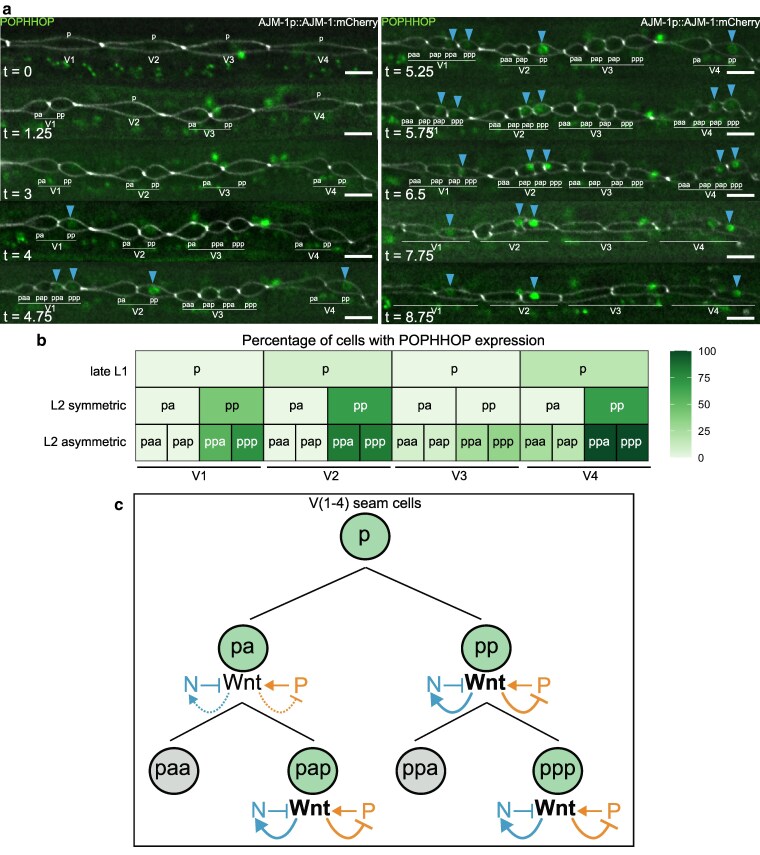
Daughter cells resulting from symmetric division show differential Wnt activation capacity. a) Representative images of POPHHOP reporter signal form late L1 (*t* = 0 h) to late L2 stage (*t* = 8.75 h). The reporter drives NLS-GFP expression, resulting in nuclear GFP signal. Time points (*t*) are indicated in hours. The membrane of the seam cells is labeled using the apical junction marker *ajm-1p::ajm-1::mCherry.* Blue arrows point to cells with GFP expression. Scale bars are 10 μm. b) Heatmap showing percentage of cells with GFP expression in V1 to V4 lineages at late L1 (p) and following the L2 symmetric (pa and pp) and asymmetric divisions (paa, pap, ppa, ppp). Cells in the posterior branch of the lineage (pp, ppa, and ppp) show stronger POPHHOP expression compared with cells in the anterior branch (pa, paa, and pap). GFP-positive cells were scored visually based on detectable nuclear signal. *n* = 12 animals. c) Schematic representation of proposed feedback regulation within the Wnt signaling pathway across symmetric and asymmetric seam cell divisions. Wnt signaling activation is hypothesized to regulate the transcription of both negative (N) and positive (P) pathway components. Upregulation of negative regulators and repression of positive regulators may act as feedback mechanisms to attenuate pathway activity following fate specification, contributing to robust asymmetric divisions. Following the symmetric division, posterior daughter cells are proposed to exhibit stronger Wnt-dependent feedback compared with anterior daughters, consistent with higher Wnt signaling activity in these cells. Green indicates seam cell fate, while gray denotes differentiating cells.

The differential expression of Wnt components in the anterior and posterior branches within each lineage might reflect differences in Wnt signaling pathway activation between daughter cells. To test this hypothesis, we utilized the POPHHOP reporter, a Wnt-responsive transcriptional reporter that serves as a readout of Wnt signaling activation, but had not been imaged before in the context of dividing seam cells (Bhambhani et al. 2014; Katsanos et al. 2017). We performed time-lapse imaging using microfluidic chambers to track POPHHOP reporter expression dynamics (Berger et al. 2021). Time-lapse imaging analysis spanning from late L1 (*t* = 0 h) through late L2 (*t* = 8.75 h) revealed dynamic POPHHOP expression patterns (Fig. 7a). At late L1, POPHHOP expression was detectable in p cells. Following the L2 symmetric division, pp cells showed higher POPHHOP activity compared with pa cells in 58% of pairs visualized. Consistent with this, cells derived from the posterior branch (ppa and ppp) displayed higher POPHHOP expression frequencies than those from the anterior branch (paa and pap) in 68% of cases (Fig. 7b). Given the high stability of the GFP reporter and the short time interval between divisions, GFP signal detected in later daughter cells is likely to reflect inheritance from the pp cell where activation is higher than pa. Taken together, these results suggest that differential expression of Wnt pathway components may be associated with differences in Wnt signaling pathway activity between pa and pp daughters following symmetric division, mediated by feedback mechanisms.

## Discussion

Comprehensive analysis of mRNA distribution patterns of Wnt pathway components following L2 symmetric and asymmetric division revealed enrichment for the negative regulators *pry-1* and *apr-1* in posterior daughter cells and enrichment for the positive regulators *sys-1*, *wrm-1*, and *lit-1*, along with the transcription factor *pop-1* in anterior daughter cells. These expression patterns are intriguing because they appear counterintuitive in light of our current understanding of Wnt pathway activation in the context of the Wnt asymmetry model. Some Wnt pathway components such as POP-1 and WRM-1 have been previously shown to be asymmetrically localized even during symmetric divisions (Wildwater et al. 2011; Hughes et al. 2013; Harandi and Ambros 2015; van der Horst et al. 2019). We have also previously shown that LIT-1 is enriched in posterior daughters following symmetric division (Ferrando-Marco and Barkoulas 2025). Interestingly, we find that in almost all cases, the expression of Wnt machinery differs in the daughter cells following symmetric division and that protein accumulation between daughter cells broadly matched the transcript patterns. For example, PRY-1 is enriched in the cytoplasm of posterior (pp) daughter cells, which leads to increased membrane and cytoplasmic PRY-1 in the daughters of the posterior branch (ppa and ppp). This branch-specific increase in PRY-1 abundance after division should be distinguished from the previously reported anterior cortical enrichment of PRY-1 in dividing mother cells (Mizumoto and Sawa 2007a, 2007b), which reflects subcellular polarity at an earlier stage rather than differences in total protein levels between daughter cells. Moreover, the *pop-1* mRNA pattern is consistent with the known protein localization. POP-1 is a transcription factor whose activity is primarily controlled by nuclear export upon phosphorylation (Meneghini et al. 1999; Rocheleau et al. 1999; Lo et al. 2004; Yang et al. 2011). Since this phosphorylation is favored in posterior daughter cells, this leads to anterior nuclear enrichment (Calvo et al. 2001; Shetty et al. 2005; Phillips et al. 2007), which is consistent with *pop-1* mRNA being enriched in anterior daughters following asymmetric division.

We reasoned that the transcriptional patterns reported here might reflect feedback within the Wnt signaling pathway. For example, Axin is a known target of Wnt signaling in vertebrates and *Drosophila* (Lustig et al. 2002; Jho et al. 2002), and in *C. elegans*, *pry-1* is upregulated upon Wnt signaling activation (Jackson et al. 2014; Gorrepati and Eisenmann 2015). Consistent with this, *pop-1(hu9)* hypomorphic animals showed altered *pry-1* transcriptional asymmetry following the L2 symmetric division. Furthermore, we also found that *pop-1* asymmetric expression is disrupted when *egl-18*, which is a POP-1 target (Gorrepati et al. 2013; Gorrepati and Eisenmann 2015), is downregulated. Transcriptional regulation of T cell factor/lymphoid enhancer factor family (TCF/LEF) by the Wnt signaling pathway has been described before. For instance, in colon cancer cells, LEF1 transcription is activated by β-catenin/TCF complexes (Hovanes et al. 2001; Li et al. 2006; Cadigan and Waterman 2012). Similarly, β-catenin and TCF4 activate TCF1 expression in intestinal epithelial cells (Roose et al. 1999). However, in human embryonic kidney cells, Wnt-dependent regulation of LEF1 expression has been shown to occur independently of TCF/LEF-β-catenin complexes, possibly involving other Wnt-induced transcription factors (Filali et al. 2002). The transcriptional asymmetries of Wnt components reported here may thus reflect both pathway-dependent regulation as well as additional inputs from cell fate determination programs acting in parallel.

Feedback regulation might extend to other members of the Wnt signaling pathway. For example, we have previously described differential expression of Wnt receptors and *Dishevelled* between the daughter cells following symmetric and asymmetric divisions, with *lin-17* being enriched in posterior daughters and *cam-1*, *mom-5* and *dsh-2* being enriched in anterior daughters, and these expression patterns could potentially also reflect feedback regulation (Ferrando-Marco et al. 2026). Both positive and negative regulation of Wnt receptors has been reported in *C. elegans* in the case of Q neuroblasts, where Wnt signaling promotes *lin-17* expression while it represses *mom-5* expression (Ji et al. 2013). Regulation of Frizzled receptor expression by Wnt signaling has also been described in other systems, including *Drosophila* and human embryonic stem cells, suggesting that receptor-level feedback is a common feature of Wnt pathway regulation (Cardigan et al. 1998; Sato et al. 1999; Willert et al. 2002).

What is the need for transcriptional feedback? The feedback in the Wnt pathway may help ensure robust asymmetric divisions, in line with the pervasive role of feedback regulation in biological systems (Masel and Siegal 2009; Whitacre 2012; Félix and Barkoulas 2015). For example, enrichment of a negative regulator such as PRY-1/Axin in posterior cells may help attenuate pathway activity after initial fate specification, preventing excessive signaling. Similarly, transcriptional repression of positive regulators such as β-catenins in posterior daughter cells following asymmetric division may also contribute to priming the system for subsequent divisions. Successive posterior enrichment and accumulation of β-catenin in asymmetric divisions has been discussed as being difficult to reconcile with Wnt signaling activity repression in anterior daughter cells (Lam and Phillips 2017). Transcriptional repression in posterior daughters might be important to limit SYS-1 and WRM-1 levels ensuring that when cells subsequently divide asymmetrically, their anterior daughters inherit lower levels of β-catenin protein, facilitating efficient degradation by the destruction complex. This transcriptional mechanism would complement posttranslational regulatory mechanisms, including centrosome localization and cell cycle–coupled degradation, to ensure robust asymmetric cell fate decisions (Vora and Phillips 2015).

We report distinct capacities for Wnt signaling activation between symmetric division daughters indicating that pa and pp cells are not identical despite adopting the same fate. Differences in Wnt pathway activation following the symmetric division are likely to arise from the preestablished polarity in the mother cell, which leads to asymmetric inheritance of Wnt activation potential in its daughters. Interestingly, because high Wnt cells show enrichment of negative regulators whereas low Wnt cells show enrichment of positive regulators, the observed mRNA distributions are more likely to result from distinct Wnt pathway activation in the 2 daughter cells and associated feedback mechanisms, rather than the reverse. It is possible that the molecular heterogeneity between the anterior and posterior branches of a lineage has functional consequences in the context of its behavior to genetic or environmental perturbations. For example, it has been reported that the anterior branch shows increased sensitivity to temperature and mutations (van der Horst et al. 2019; Hintze et al. 2020). Regardless of the functional impact, these findings suggest that seam cell lineages may be less homogeneous than previously thought, with evident molecular asymmetry in the daughter cells from a seemingly symmetric division, which is important to be considered in the study and interpretation of developmental phenotypes.

## Supplementary Material

iyag147_Supplementary_Data

## Data Availability

All strains and smFISH probes used in this study are described in Supplementary Table 1 and are available upon request. All data necessary for confirming the conclusions of the work are present within the article and figures. All statistical analyses supporting the findings are presented in Supplementary Table 2. Supplemental material available at GENETICS online.
