# Supplementary material for: Transcriptional feedback targeting Wnt pathway components reveals hidden heterogeneity in Caenorhabditis elegans seam cell lineages
Source: Genetics. 2026 Jun 12;233(4):iyag147. doi: 10.1093/genetics/iyag147 (PMC13439692; doi:10.1093/genetics/iyag147)
Supplement: Supplementary file 1 [file iyag147_supplementary_data.zip › Supplemental_Data_and_Figure_S1_GENETICS-2026-309427.pdf]

Supplementary Data

Transcriptional feedback targeting Wnt pathway components reveals hidden heterogeneity in *C. elegans* seam cell lineages.

Mar Ferrando-Marco[1], Simon Berger[2] and Michalis Barkoulas[1]

[1]Department of Life Sciences, Imperial College, Exhibition Road, London SW7 2AZ, United Kingdom.

[2]Department of Molecular Life Sciences, University of Zurich, Winterthurerstrasse 190, 8057 Zurich, Switzerland

Figure S1: Analysis of *pry-1* and *wrm-1* active transcription events.

Table S1: Strains and smFISH probes used in this study.

Table S2: Extended statistical analysis for all datasets presented in this study.

a

pry-1 smFISH

Cell	p	pa	pp	paa	pap	ppa	ppp
% of cells with intense spots	24%	0%	61%	1%	43%	0%	37%
Total number of cells	38	84	84	73	73	73	73
Fisher's exact test	N/A	$p < 0.001$		$p < 0.001$		$p < 0.001$	

b

wrm-1 smFISH

Cell	p	pa	pp	paa	pap	ppa	ppp
% of cells with intense spots	30%	12%	3%	5%	0%	8%	0%
Total number of cells	67	78	78	73	73	73	73
Fisher's exact test	N/A	$p = 0.05$		$p = 0.11$		$p = 0.02$	

Figure S1. **Analysis of *pry-1* and *wrm-1* active transcription events**. **(a-b)** Percentage of cells containing intense nuclear spots that might correspond to active transcription events in *pry-1* smFISH (a) and *wrm-1* smFISH (b).

Fisher's exact *p*-value was calculated between anterior (pa, paa, ppa) and posterior (pp, pap, ppp) lineages for each developmental stage, respectively. p = late L1, pa, pp = L2 symmetric, paa, pap, ppa, ppp = L2 asymmetric.
